# Suggested randomised, controlled trial with frovatriptan

**DOI:** 10.1007/s10194-011-0381-x

**Published:** 2011-09-14

**Authors:** Peer Tfelt-Hansen, Timothy J. Steiner

**Affiliations:** 1 Danish Headache Center, Glostrup Hospital, University of Copenhagen, Glostrup, Denmark; 2 Norwegian Headache Centre, St Olavs Hospital, Norwegian University of Science and Technology, Trondheim, Norway

Sir,

**Frovatriptan versus other treatments for acute migraine**

There are now three crossover, double-blind, randomized controlled trials (RCTs) of similar design comparing frovatriptan 2.5 mg with zolmitriptan 2.5 mg [1], rizatriptan 10 mg [2] and almotriptan 12.5 mg [3]. Each RCT found no significant differences between the treatments with regard to patients’ expressed preferences and, as secondary outcome measures, the conventional end-points of pain-freedom and headache relief [4]. A meta-analysis of the three trials, all without placebo comparison, found them to be the sufficient evidence of equal efficacy of frovatriptan and these other triptans [5].

Is this useful knowledge? There appears to be a consensus supporting head-to-head comparisons between triptans as the evidential basis for selecting best treatment. Clinical experience, which is not easily converted into formal evidence, nonetheless demonstrates that differences between triptans at group level are generally less influential than the differences in individual responses to them. So, in practice, those for whom triptans are indicated will still need to make their own comparative assessments.

The prior question is: for whom are triptans indicated? Accordingly, one important comparative RCT is missing. Effervescent aspirin 1,000 mg was quite similar to sumatriptan 50 mg in the treatment of migraine attacks in a meta-analysis of three RCTs [6]. The World Health Organization currently advocates only aspirin or paracetamol as treatments for acute migraine in adults [7]. It would be of great help to people with migraine worldwide if triptans were shown to be superior to aspirin.

The clinically relevant RCT is therefore frovatriptan 2.5 mg versus effervescent aspirin 1,000 mg with the crossover design and, ideally, placebo control [4].

Yours sincerely

Peer Tfelt-Hansen, MD, DMSc

Timothy J Steiner, MB, PhD, LLM
